# The Effects of Landscape Variables on the Species-Area Relationship during Late-Stage Habitat Fragmentation

**DOI:** 10.1371/journal.pone.0043894

**Published:** 2012-08-24

**Authors:** Guang Hu, Jianguo Wu, Kenneth J. Feeley, Gaofu Xu, Mingjian Yu

**Affiliations:** 1 The Key Laboratory of Conservation Biology for Endangered Wildlife of the Ministry of Education, College of Life Sciences, Zhejiang University, Hangzhou, People’s Republic of China; 2 School of Life Sciences & Global Institute of Sustainability, Arizona State University, Tempe, Arizona, United States of America; 3 Sino-United States Center for Conservation, Energy, and Sustainability, Inner Mongolia University, Hohhot, People’s Republic of China; 4 Department of Biological Sciences, Florida International University, Miami, Florida, United States of America; 5 Center for Tropical Plant Conservation, Fairchild Tropical Botanic Garden, Coral Gables, Florida, United States of America; 6 Xin’an River Development Corporation, Chun’an, People’s Republic of China; Duke University, United States of America

## Abstract

Few studies have focused explicitly on the later stages of the fragmentation process, or “late-stage fragmentation”, during which habitat area and patch number decrease simultaneously. This lack of attention is despite the fact that many of the anthropogenically fragmented habitats around the world are, or soon will be, in late-stage fragmentation. Understanding the ecological processes and patterns that occur in late-stage fragmentation is critical to protect the species richness in these fragments. We investigated plant species composition on 152 islands in the Thousand Island Lake, China. A random sampling method was used to create simulated fragmented landscapes with different total habitat areas and numbers of patches mimicking the process of late-stage fragmentation. The response of the landscape-scale species-area relationship (LSAR) to fragmentation *per se* was investigated, and the contribution of inter-specific differences in the responses to late-stage fragmentation was tested. We found that the loss of species at small areas was compensated for by the effects of fragmentation *per se,* i.e., there were weak area effects on species richness in landscapes due to many patches with irregular shapes and high variation in size. The study also illustrated the importance of inter-specific differences for responses to fragmentation in that the LSARs of rare and common species were differently influenced by the effects of fragmentation *per se*. In conclusion, our analyses at the landscape scale demonstrate the significant influences of fragmentation *per se* on area effects and the importance of inter-specific differences for responses to fragmentation in late-stage fragmentation. These findings add to our understanding of the effects of habitat fragmentation on species diversity.

## Introduction

Habitat fragmentation, which is widely recognized as one of the leading threats to biodiversity, consists of two simultaneous processes: 1) the loss of habitat area and 2) the division of habitat into isolated patches [1]–[4]. In this paper, we refer to this second process of changes in spatial configuration of habitat as “fragmentation *per se*”, distinct from “habitat fragmentation” which is the combination of area loss and change of spatial configuration. While many studies have focused on the effects of area loss on species diversity, the effects of fragmentation *per se*, and the associated changes in the spatial arrangement and configuration of habitat patches within landscapes on species diversity have received considerably less attention. Actually, fragmentation *per se* can result in smaller patches and other associated changes (e.g. reduction in core habitat and connectivity; increases in patch number, total edge, perimeter-area ratio and shape complexity) by the breaking apart of continuous habitat into fragments, which also can compound or exasperate the effect of habitat loss. In the past few decades, some studies have explored the effects of fragmentation *per se* using statistical methods [5], [6] and experimental approaches [7]–[10]. However, these studies have generally produced contradictory results. In some studies, species diversity and population density increased due to habitat fragmentation *per se* both at the patch scale and the landscape scale, due to the “crowding” effect where surviving individuals move from the disturbed habitat matrix into the remaining fragments [8], and/or increased habitat heterogeneity [9]. In contrast, some studies have found negative effects of fragmentation *per se* on species diversity due to higher rates of local extinction [5], [6] and negative edge effect [7]. One possible explanation for these variable results is that the effects of habitat loss and fragmentation *per se* in real landscapes are difficult to separate because of the complex influence of different biotic and abiotic factors, potentially confounding smaller-scale experiments or analyses. Likewise, the statistical methods commonly employed in these previous studies have been questioned because of the difficulty in distinguishing the effects of habitat loss vs. fragmentation *per se* [11], [12].

**Figure 1:**
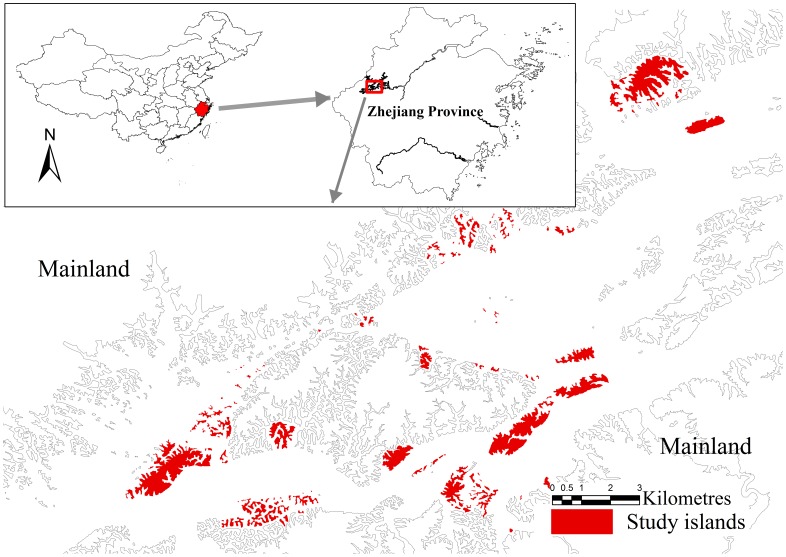
The 152 study islands in the Thousand Island Lake, China.

The effects of habitat loss on biodiversity are typically considered to be stronger than the effects of fragmentation *per se* [13], [14], [15]. However, this generally assumes that during the process of fragmentation there is a monotonic decrease in habitat area and increasing fragmentation *per se* [16], [17]. Contrary to this assumption, several studies have found that the landscape attributes related to fragmentation *per se* (such as patch number, mean patch size and patch size variability) change unimodally over the entire process of habitat fragmentation [18], [19]. During the later stage of habitat fragmentation, or “late-stage fragmentation”, both total habitat area and patch number decline. Consequently, the responses of species diversity to area loss and fragmentation *per se* and their combined effects are likely to be complex and dependent on the actual patterns of habitat fragmentation and degree of habitat loss [4], [20].

It is also important to recognize that habitat fragmentation is a landscape-scale process but that most previous studies of fragmentation have focused on patch-scale patterns or phenomena [1]. Small sample sizes and a dichotomous characterization of habitats (continuous or fragmented) may bias the estimation of fragmentation effects and may overlook important landscape-level features that can be key determinants of species diversity [20], [21], [22]. As such, the effects of fragmentation *per se* as related to spatial configuration can only be tested at the landscape scale [1].

**Figure 2:**
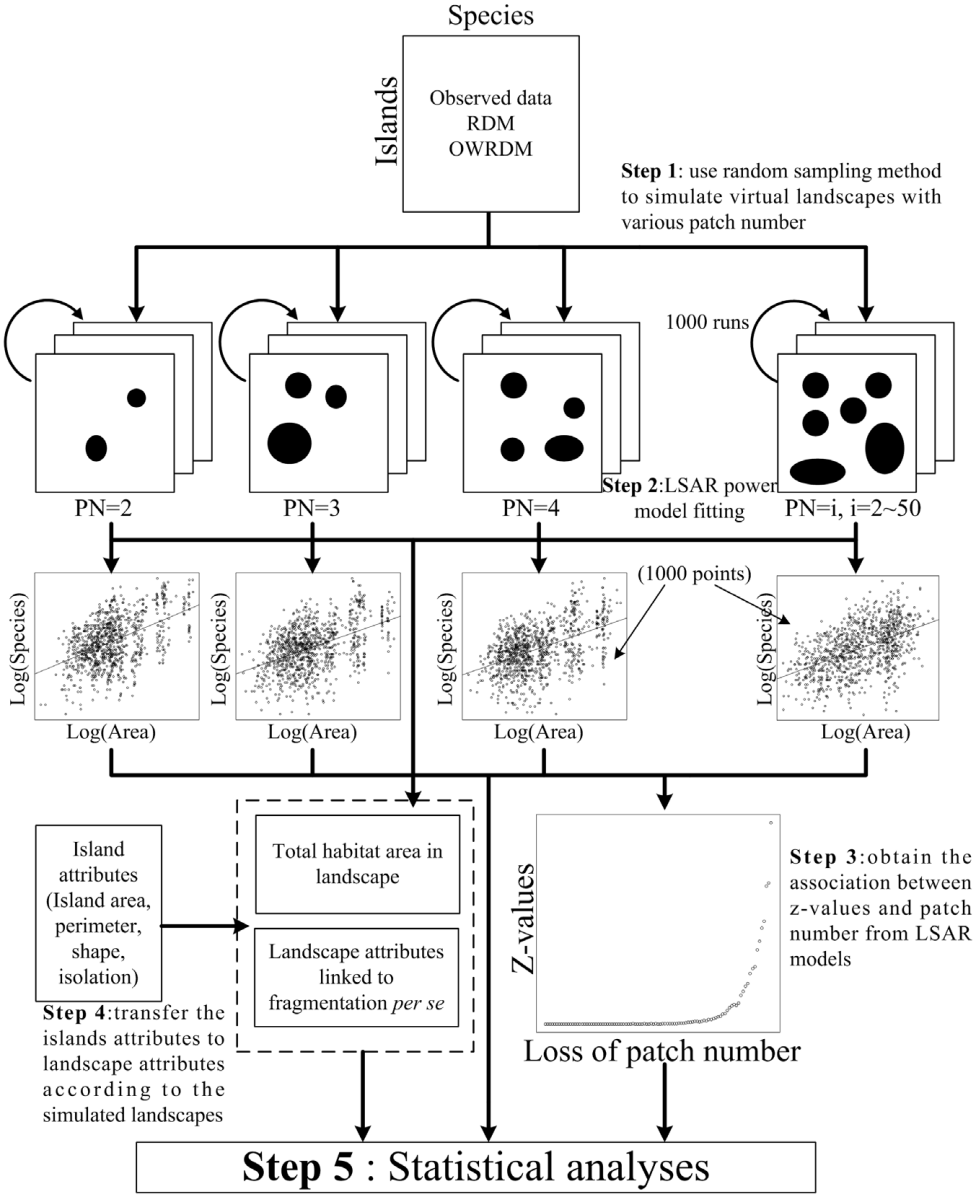
The conceptual diagram indicating the main process of model simulation and analyses used in this study.

The positive relationship between habitat area and species diversity, or the Species-Area Relationship (SAR), is one of the most important phenomena in ecology and has been frequently used to describe the effects of area loss on species diversity [23], [24], [25], [26]. Typically, plotting number of species against the sampling area, within a set of samples of increasing areas exhibits a monotonically increasing curve whose slope is firstly steep but becomes nearly flat later [23], [24]. Indeed, many studies of fragmented landscapes have found strong area effects on species diversity and concluded that differences in habitat area is the primary factor determining patterns of species diversity [27], [28], [29]. Meanwhile, it is often overlooked that landscape-scale attributes related to fragmentation *per se* could also be significantly affecting the observed SARs in fragmented landscapes [30], [31].

Not all species show comparable responses to habitat fragmentation. Species with greater tolerances to habitat fragmentation can become abundant and widespread, while more “sensitive” species decrease in abundance or only persist within restricted subsets of fragments [32]. For example, previous studies investigating the relationship between functional traits and local species richness have indicated that species preferring core habitats had higher rates of local extinction in fragmented landscape than habitat generalists or species that tolerate/prefer edge habitat [29], [33], [34]. Consequently, the occurrence of individual species across fragments (i.e., the proportion of fragments where a species occurs) can be used to estimate their vulnerability to local extinction caused by habitat fragmentation [34]. Furthermore, differences in species occurrence patterns can affect overall patterns of species richness. Sizling *et al.* [35] found that common species have a stronger influence than rare species on species richness patterns. Thus, separately comparing the effects of landscape attributes on rare vs. common species may provide useful information and help to increase our understanding of the mechanisms through which habitat fragmentation affects species diversity.

**Table 1:** Spearman’s correlation coefficients among six landscape attributes in the model simulations created from observed species richness data (PN: patch number; PSV: patch size variability; LSI: landscape shape index; MDM: mean nearest distance to mainland).

	Area	PN	PSV	LSI
PN	0.043***			
PSV	0.541***	0.249***		
LSI	0.152***	0.909***	0.142***	
MDM	−0.083***	0.156***	0.063***	0.086***

*** *P*<0.001.

**Table 2:** Partial correlation coefficients between landscape attributes and observed species richness/*z*-values as calculated over the entire process of late-stage fragmentation.

Attributes	Controlling for PN		Controlling for Area	
	Richness	Z-value	Richness	Z-value
Area	0.412***	0.005^NS^	–	–
PN	–	–	0.917***	−0.436***
PSV	0.257***	−0.198***	0.262***	−0.326***
LSI	0.125***	−0.021^NS^	0.840***	−0.407***
MDM	0.052***	−0.043***	0.185***	−0.108***

The abbreviations are the same as in Table 1.

NS, *P*>0.05;

*** *P*<0.001.

The Thousand Island Lake (TIL) is a man-made reservoir in East China (inundated in the 1950’s) that has >1000 islands that were created out of erstwhile hilltops during inundation. In this study, we investigated plant species richness and composition on 152 of the TIL islands in order to examine how fragmentation *per se* can affect species richness and landscape-scale SARs during late-stage fragmentation. Specifically, we addressed two questions: (1) How does fragmentation *per se* influence the effects of area loss on diversity at the landscape scale? and (2) Do the effects of fragmentation *per se* differ between rare and common species?

## Materials and Methods

### Study Site

Our study was conducted across a land-bridge island system in the Thousand Island Lake (TIL; Fig. 1). TIL is located in Zhejiang Province in China (29°22′–29°50′ N and 118°34′–119°15′ E) and was formed in 1959 when the Xin’an River was dammed for the purpose of generating hydroelectricity. The rising water inundated an area of 573 km^2^ in a topographically complex landscape resulting in the formation of 1078 land-bridge islands with areas ranging from approximately 0.25 to 1320 ha. Before dam construction and the simultaneous emigration of local people, the forests on the hills were clear-cut. Since inundation, the lake has been protected by law and the vegetation on the islands (erstwhile hilltops) has not experienced significant human disturbance. Currently, these islands are covered primarily by secondary pine forests with canopy composition dominated by *Pinus massoniana*. The TIL region has a subtropical monsoon climate with an average annual temperature of 17.0°C, ranging from −7.6°C in January to 41.8°C in July. The average annual precipitation in the region is 1430 mm.

**Figure 3:**
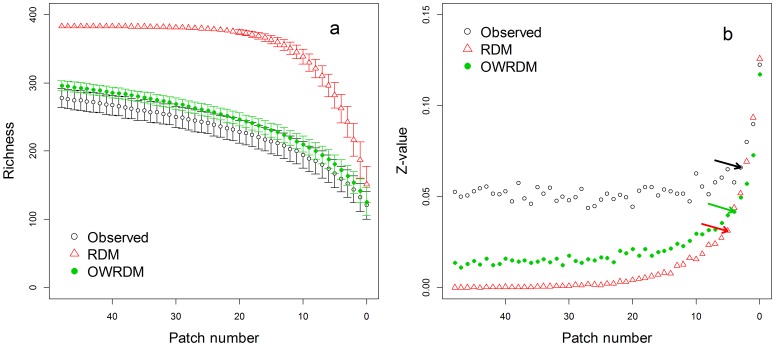
The responses of (a) richness and (b) *z*-values to number of habitat patches using three datasets (observed, RDM and OWRDM). Error bars showed the standard deviations of richness. The arrows pointed out the calculated threshold location.

**Figure 4:**
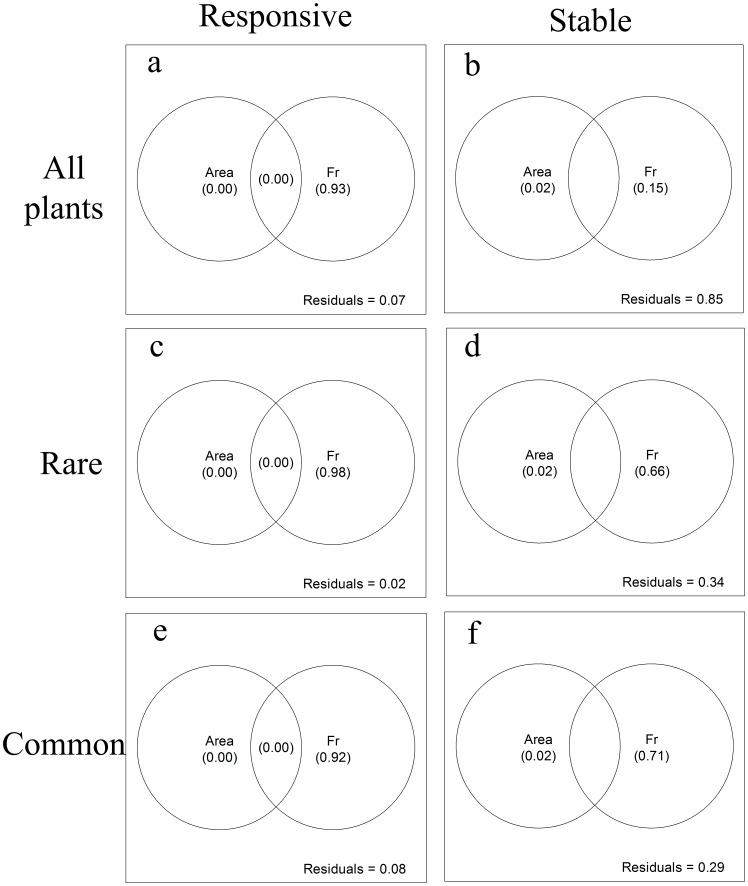
Variation partition of landscape attributes for z-values of LSARs for (a, b) all plants, (c, d) rare species and (e, f) common species during the responsive phase and the stable phase. The independent and combined explanatory of each variable showed in brackets (values<0 are not shown). (Fr: fragmentation *per se*, including PN, PSV, LSI and MDM).

Man-made land-bridge islands formed by the damming of rivers, such as those of TIL, have been referred to as ideal “experimental” or model systems for studying habitat fragmentation [36]–[39]. This is because they have a homogeneous matrix, distinct habitat boundaries, and are formed by a single simultaneous disturbance event. This combination of features minimizes the influence of several confounding factors related to disturbance history, succession, and matrix heterogeneity that have caused difficulties in previous studies of terrestrial fragmented landscapes [2], [40], [41].

### Data Collection

During the growing seasons of 2007 and 2008, we conducted vegetation surveys on 152 islands in TIL. During the surveys, we determined the occurrence of all plant species (i.e., presence/absence - abundance data were not collected) through multiple visits to all islands and using an area-dependent proportional sampling procedure [42] designed to record the maximum possible number of species. Specifically, each of the study islands <50 ha were circumnavigated and 4–16 transects with 5 m width were established (total length of transects were dependent on a logarithmic scale, assuming a SAR with a slope (z) of 0.16 in a log-log scale as based on our previous studies [29]: placing 400 m of transects on the islands ≤1 ha in area, 800 m of transects on 10 ha islands, 1600 m of transects on 100 ha islands and so on). Each transect was walked a minimum of three times by trained observers. For the three study islands >100 ha in area, surveys were conducted as above but at multiple points per island centered on each prominent hill. Species accumulation curves indicate that these methods were sufficient to capture all or most species present on the islands [43]. Most plant species, including herbs and ferns, were identified and recorded in the field. Unidentified specimens were collected and identified in the lab with the assistance of taxonomic experts and available literature [44], [45]. All plant species were divided into rare and common species according to their patch occupancy. Namely, species that were recorded on ≤10% of the study islands (i.e., 15 islands) were classified as “rare” and species that occurred on >10% of the study islands were classified as “common”.

**Figure 5:**
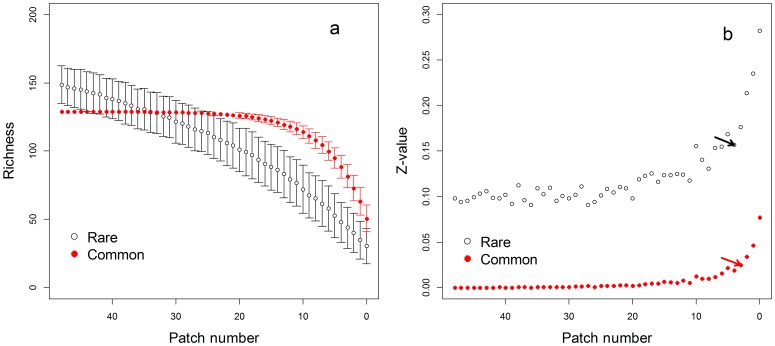
The responses of (a) species richness and (b) *z*-values of rare and common species to patch loss. Error bars showed the standard deviation. The arrows pointed out the threshold location.

Patch area and perimeter of 152 islands (Table S1) were calculated through analysis of paper maps digitized into ArcGIS 9.3 [46] assuming a water level of 105 m.a.s.l. (generally corresponding to the edge of forest cover; in calculating the island area and in all subsequent analyses we excluded any beach that may form below this level during periods of low water.). We measured the landscape habitat area using the total land-surface area across the study islands and estimated the degree of fragmentation *per se* using patch number (PN = number of patches in the landscape), patch size variability (PSV = the coefficient of variation of patch area), landscape shape index (LSI), and mean distance from islands to the mainland (MDM). LSI indicated the relative shape complexity in the landscape and was calculated as LSI = E/minE, where E was the total length of edge in landscape and minE was the minimum total length of edge in landscape (the perimeter of a circle with the area equaling to the total landscape area) with vector data [47]. MDM was calculated as the shortest linear distances from island edge to mainland edge. PSV and LSI reflected the degree of habitat diversity and MDM reflected the average degree of habitat isolation of the landscape.

### Data Analysis

The power law (*S = cA^z^*, where *S* is species richness, *A* is area, *c* is a region-specific constant, and *z* is the scaling exponent), was used in our analyses of area effects [48], [49], [50]. In this study, we focused on the landscape-scale SAR (LSAR), which is different from the typical SAR which is calculated at the patch scale. The LSAR regression model uses the log-transformed total species richness for each landscape calculated by pooling the observed species lists of their constituent islands as the response variable and the log-transformed total land-surface area for each landscape (i.e., the summed area of all islands in the landscape) as the predictor variable. As such, the LSAR *z*-value reflects the sensitivity of the species diversity within an entire landscape to changes in total habitat amount [42], [51] and can be used to examine the influence of changes in landscape-level attributes associated with fragmentation *per se*.

In addition to the observed species presence/absence data, we also generated two types of null datasets using a random distribution model (RDM) and a species-occurrence weighted RDM (OWRDM). For the RDM, the observed species richness was retained on each island but the identities of the occurring species were selected at random without replacement from a finite species pool (the complete plant species list across all 152 islands). The sampling process was repeated independently for each island. To incorporate inter-specific difference into this random model, we also modified the stochastic sampling process to construct the OWRDM in which the probability of species selection in the sampling process was weighted by the observed relative occurrence of each species at the landscape scale (measured as the proportion of the study islands where the focal species was recorded).

To simulate the process of habitat fragmentation and the associated changes in the number of patches in the landscape, we used a random sampling method to generate 49 simulated landscape configurations with 2 to 50 patches (Fig. 2) and assuming that the effects on diversity are mainly associated with changes in degree of isolation and patch characteristics and not substantially influenced by the configuration of the surrounding patches. The patches in these simulated landscapes were drawn randomly without replacement from the 152 study islands in TIL. The range of total habitat area in each simulated landscape was limited from 11.85 ha (minimal total area of the landscape with 50 patches) to 259.12 ha (maximal total area of the landscape with 2 patches) to confirm that the area distribution of each landscape could overlap each other. To make the resampling points of the total area of simulated landscapes evenly distributed within the limited range, we then divided the range into five isometric sections, and iterated this process 200 times to create replicate landscapes within each section and for each number of patches. Thus, we created a total of 49000 (200×5×49) simulated landscapes with relatively homogeneous numbers of points across area for each number of patches in our analyses. We then applied the LSAR model to the replicates to derive a landscape-level *z*-value for each set of simulated landscapes with a given number of patches. Next, the relationships between the landscape *z*-values and other variables derived for each of the simulated landscapes were used to estimate the dependence of LSAR model to fragmentation *per se*. We used Spearman’s rank correlation analysis to test the interaction among landscape variables based on data derived from the simulated landscapes. The partial Spearman correlation controlling for the effect of area and number of patches was applied to test the effects of the landscape variables (area, PN, PSV, LSI and MDM) respectively on species richness and *z*-values of LSARs derived from observed dataset. We used the pcor.test () function in R package {ppcor} [52] to run the multiple correlation tests. In addition, the variation partition method based on the redundancy analysis [53], [54] was used to determine the relative contributions of area loss and fragmentation *per se* (the combination of PN, PSV, LSI and MDM) to the variation in *z*-values with the observed data set. The method was implemented using the varpart () function in R package {vegan} [55]. The significance levels of the above analyses were adjusted by Bonferroni correction. We identified the location of possible thresholds for changes in the relationships between the *z* values and the number of patches by piecewise regression [56] using the piecewise.linear () function in the R package {SiZer} [57]. If a threshold exists in the relationships, it can be used to divide the process of late-stage fragmentation into two distinct phases: the “stable phase” during which there is little change in the *z*-values with changes in the number of patches and the “responsive phase” during which z-values change rapidly with changes in the number of patches. We also applied the same statistical methods to analyze patterns for rare vs. common species and their responses to fragmentation *per se* in the stable and responsive phases. All statistical analyses were performed using R 2.10.1 [58].

## Results

We recorded 383 species of vascular plants on the 152 study islands in the TIL during the growing seasons of 2007 and 2008 (Table S2, S3). According to the classification criteria described above, 254 of the species were considered “rare” and 129 were considered “common”.

Area, PN, PSV and LSI were all significantly positively correlated with each other. All variables except MDM were significantly negatively correlated with area in the observed dataset (Table 1). During late stage-fragmentation, partial correlation analysis showed that area was significantly correlated to species richness, but not correlated with *z*-values when we controlled for patch number (Table 2). The partial correlation also showed that the number of patches was positively correlated with species richness but negatively correlated with *z*-values even when controlling for area. The other landscape variables mostly showed positive correlations with richness and negative correlations with z-values (Table 2). But LSI was not significantly correlated with *z*-values when controlling for patch number.

In the comparison between the observed data set and the two simulated data sets (RDM and OWRDM), there were no significant differences in species richness in the observed data and simulated OWRDM datasets (Fig. 3a). In the three data sets, the *z*-values vs. patch number relationships exhibited significant thresholds at similar locations as indicated by piecewise regression (observed data, PN = 5, R^2^ = 0.88, *p*<0.001; RDM, PN = 7, R^2^ = 0.96, *p*<0.001; OWRDM, PN = 6, R^2^ = 0.93, *p*<0.001). Differing from the other datasets’ performances in the stable phase, the *z*-values derived from RDM approximated zero (Fig. 3b). The *z*-values of OWRDM were closer to that derived from the observed data than the z-values of RDM to observed data. In the responsive phase, the *z*-values of the three data sets all increased rapidly with decreasing number of patches. By variation partition analysis, fragmentation *per se* was a principle main factor explaining the variation in *z*-values during the responsive phase (93%, Fig. 4a). No factor could explain the variation of *z*-values in the stable phase (unexplained 85%, Fig. 4b).

Rare species richness decreased consistently with decreasing number of patches (Fig. 5a). In contrast the richness of common species exhibited a “hockey stick” pattern with distinct stable and responsive phases. Despite obvious differences between the responses of rare and common species richness, the *z*-values of both exhibited threshold-like patterns (Fig. 5b; piecewise regression: rare species, PN = 7, R^2^ = 0.96, *p*<0.001; common species, PN = 6, R^2^ = 0.93, *p*<0.001). The pattern of common species’ *z*-values (Fig. 5b) was similar to that of RDM (Fig. 3b). But the pattern of rare species’ *z*-values resembled that derived from the observed data of all species. Variation partition analysis illustrated that variation in area effect for rare species was also explained by the effect of fragmentation *per se* during the two phases (Fig. 4c, d). During the responsive phase, fragmentation *per se* independently explained 92% of the variation in *z*-values for common species (Fig. 4e), and also explained 71% of the variation in the stable phases (Fig. 4f).

## Discussion

Habitat amount, patch size, number of patches and connectivity are four basic descriptors of fragmented landscapes [59]. Often, habitat amount and number of patches are inversely correlated as increasing habitat fragmentation results in more patches but less total habitat area [1], [4], [17], [59]. This inverse relationship between habitat area and number of patches has complicated and potentially confounded previous studies looking at the effects of fragmentation *per se* on species diversity. In the present study, we overcame this difficulty by focusing exclusively on late-stage fragmentation during which the total area of habitat in the landscape and the number of habitat patches both decrease due to the elimination of existing patches.

We found a positive relationship between landscape-level species richness and total habitat area at TIL. This relationship is consistent with the results of other studies at TIL conducted at the patch scale [29], [43], [60] and may be caused by rising extinction rates and decreasing habitat heterogeneity (i.e., landscapes containing more patches with irregular shapes and various sizes can support more species than simpler landscapes with fewer patches). The significant correlations between PSV/LSI and richness are also consistent with this explanation. Species richness was also associated with MDM, the landscape attributes related to connectivity and isolation. This may be due to the limited dispersal of some species from mainland to islands. Supporting this, previous studies at TIL have found that isolation affected the species composition and nestedness of plant communities on individual islands [29], [43].

The landscape variables PN, PSV, LSI and MDM all influenced the relationships between habitat area and species diversity when controlling for area effect during late-stage fragmentation (Table 2), supporting the hypothesis that patch number, habitat diversity and isolation can all influence the patterns of species diversity during this phase. Specifically, a large number of patches can provide high habitat diversity for species with different environmental requirements [61], [62] and result in decreased regional extinction risks due to larger local population sizes. More patches are also known to result in more potential connections and the stepping-stones which can increase the possibility of “rescue effect” [63]–[67].

As seen in Fig. 3b, the *z*-values of LSARs exhibited a threshold-dependent relationship with the number of habitat patches in the landscape. When the number of patches in the landscape exceeded this threshold, species richness maintained a stable decreasing rate despite changes in patch number or other landscape-level variables. But when the patch number was lower than the threshold, there was a rapid decrease in species richness and thus a steep increase in *z*-values. Additionally, we found that the *z*-values in the stable phase were much lower than the *z*-value (0.16) at the patch scale in TIL [29] and the typical range of *z*-values (0.2–0.3) in previous studies looking at patterns of species richness across fragments or islands [26], [68], [69]. The lack of an area effect in the stable phase illustrates that there is a potential compensatory effect on species richness of high PN and habitat diversity preventing the rapid loss of species diversity in late-stage fragmentation. In contrast, below the threshold (i.e., in the responsive phase), *z*-values increased rapidly and species richness decreased rapidly with the loss of patches. The significant effects of MDM on species richness and z-values indicated that it is possible that the strong effect of patch elimination during the responsive phase was due to increasing effective separation (not equal to geographical isolation) of the sparse islands, inhibiting colonization [70] and consequently weakening rescue effect. The decrease in habitat diversity with decreasing patch number may also be an important factor driving the responsive phase patterns. Another potential explanation is the sampling theory [71], which predicts that patches sampled from a landscape with few patches will contain a larger proportion of the total diversity than patches sampled from landscape with more patches.

Our results support the hypothesis that the effects of habitat fragmentation are influenced by inter-specific differences [59], [72], [73]. This is highlighted by the differences between the three datasets (observed, RDM and OWRDM, Fig. 3) which incorporated different degrees of inter-specific variation. For example, with the RDM dataset, in which all species were equivalent, *z*-values approximated zero in the stable phase due to the absence of any species-area relationship. This indicates that in the absence of inter-specific differences (i.e., different niches, different tolerances to disturbance or different functional traits), diversity may not decrease due to area loss as long as there are a sufficient number of patches in the landscape.

Furthermore, we saw different response patterns between rare and common species, consistent with the results of our previous studies [29], [43]. The area effects of rare and common species (Fig. 5b) were both more significantly affected by patch number than that of all plants (Fig. 3b, observed data). These results indicate that more significant effects of fragmentation *per se* can be found in the respective analyses of different sub-groups than in the holistic analysis of entire plant community, and this was also supported by the higher explanatory power for piecewise regression in rare and common species respectively than in all plants. For rare species, landscape-level *z*-values exhibited an increasing trend with decline of patch number even in the stable phase. This may be due to the fact that the specific habitat requirements of rare species decrease the effective patch number below the total number available in the landscape. For common species, the observed patterns of *z*-values and species richness (Fig. 5) were comparable to what was found for RDM dataset (Fig. 3) suggesting a lack of functional differences between these species.

In sum, we found the effects of fragmentation *per se* had an overcompensation effect that outweighed the effect of area within the fragmented landscapes containing more patches with irregular shapes and various sizes and enhanced the area effect in the simpler fragmented landscapes with fewer patches. Rare and common species exhibited different response to the habitat fragmentation. Thus, inter-specific difference may simultaneously influence the process of community assembly and the patterns of species diversity in fragmented landscapes.

## Supporting Information

Table S1 **Basic information of 152 study islands in the Thousand Island Lake.**
(DOCX)

Table S2 **Vascular plant species list on 152 islands in the Thousand Island Lake.**
(DOCX)

Table S3 **The species distribution on 152 study islands in the Thousand Island Lake.** The dark cell means that the species is present on the islands, and the white cell means that the species is absent on the islands. Com_sp: common species; rar_sp: rare species.
(XLSX)
