# Supplementary material for: The Effects of Landscape Variables on the Species-Area Relationship during Late-Stage Habitat Fragmentation
Source: PLoS One. 2012 Aug 24;7(8):e43894. doi: 10.1371/journal.pone.0043894 (PMC3427301; doi:10.1371/journal.pone.0043894)
Supplement: Table S1 — Basic information of 152 study islands in the Thousand Island Lake. (DOCX) [file pone.0043894.s001.docx]

Table S1 Basic information of 152 study islands in the Thousand Island Lake. DM: nearest distance from each island to the mainland, S: species number of vascular plants.

| **Island** | **Perimeter (m)** | **Area (ha)** | **DM (m)** | **S** |
| --- | --- | --- | --- | --- |
| I1 | 1033.02 | 2.01 | 856.31 | 72 |
| I2 | 651.30 | 0.98 | 1271.34 | 59 |
| I3 | 523.63 | 0.99 | 926.9 | 42 |
| I4 | 241.37 | 0.31 | 528.19 | 77 |
| I5 | 329.46 | 0.30 | 940.53 | 46 |
| I6 | 556.33 | 0.73 | 1557.26 | 85 |
| I7 | 312.95 | 0.27 | 884.39 | 76 |
| I8 | 5934.01 | 13.40 | 439.47 | 100 |
| I9 | 483.03 | 0.91 | 912.03 | 69 |
| I10 | 316.97 | 0.29 | 972.58 | 55 |
| I11 | 1184.67 | 2.17 | 998.41 | 84 |
| I12 | 573.69 | 1.44 | 1106.53 | 100 |
| I13 | 493.10 | 0.84 | 1026.58 | 69 |
| I14 | 342.83 | 0.46 | 1982.69 | 78 |
| I15 | 352.96 | 0.62 | 2333.6 | 56 |
| I16 | 368.64 | 0.43 | 36.12 | 77 |
| I17 | 1187.41 | 2.81 | 577.84 | 112 |
| I18 | 438.94 | 0.89 | 293.41 | 56 |
| I19 | 216.79 | 0.30 | 220.9 | 57 |
| I20 | 512.15 | 1.03 | 665.45 | 88 |
| I21 | 554.80 | 1.35 | 936.42 | 65 |
| I22 | 146.33 | 0.16 | 1086.59 | 68 |
| I23 | 1371.81 | 4.66 | 169.62 | 86 |
| I24 | 392.85 | 0.62 | 1378 | 68 |
| I25 | 172.17 | 0.18 | 1502.39 | 49 |
| I26 | 378.10 | 0.99 | 30.46 | 63 |
| I27 | 839.07 | 2.23 | 1149.59 | 86 |
| I28 | 539.85 | 1.30 | 466.8 | 64 |
| I29 | 207.22 | 0.18 | 1039.95 | 64 |
| I30 | 249.75 | 0.25 | 1284.56 | 74 |
| I31 | 402.52 | 0.79 | 2657.77 | 69 |
| I32 | 216.90 | 0.25 | 2658.07 | 69 |
| I33 | 274.91 | 0.36 | 2227.08 | 72 |
| I34 | 146.65 | 0.12 | 2073.07 | 45 |
| I35 | 370.60 | 0.53 | 1939.94 | 66 |
| I36 | 170.83 | 0.19 | 2137.68 | 58 |
| I37 | 583.41 | 1.32 | 2121.37 | 75 |
| I38 | 606.75 | 1.51 | 818.75 | 97 |
| I39 | 437.77 | 1.00 | 701.87 | 59 |
| I40 | 360.23 | 0.65 | 1352.59 | 67 |
| I41 | 339.48 | 0.57 | 905.96 | 54 |
| I42 | 550.14 | 1.30 | 925.05 | 67 |
| I43 | 1364.00 | 3.70 | 2225.45 | 102 |
| I44 | 803.40 | 1.33 | 2016.8 | 58 |
| I45 | 983.21 | 2.07 | 1868.45 | 56 |
| I46 | 320.07 | 0.35 | 1495.46 | 48 |
| I47 | 420.45 | 0.65 | 755.8 | 66 |
| I48 | 626.72 | 0.81 | 1049.81 | 58 |
| I49 | 190.72 | 0.21 | 2433.91 | 62 |
| I50 | 203.60 | 0.29 | 3073.21 | 42 |
| I51 | 396.92 | 0.61 | 653.07 | 59 |
| I52 | 1105.00 | 3.71 | 560.8 | 79 |
| I53 | 1053.42 | 2.29 | 493.77 | 73 |
| I54 | 194.88 | 0.16 | 1056.66 | 54 |
| I55 | 335.13 | 0.28 | 1093.74 | 48 |
| I56 | 342.78 | 0.60 | 808.92 | 55 |
| I57 | 1605.47 | 3.42 | 626.61 | 90 |
| I58 | 451.51 | 0.84 | 690.03 | 46 |
| I59 | 221.61 | 0.19 | 398.18 | 45 |
| I60 | 166.59 | 0.14 | 387.1 | 39 |
| I61 | 297.74 | 0.25 | 718.52 | 34 |
| I62 | 1108.32 | 2.37 | 1198.94 | 57 |
| I63 | 602.60 | 1.33 | 730.95 | 81 |
| I64 | 561.71 | 1.31 | 868.22 | 42 |
| I65 | 476.90 | 0.94 | 330.95 | 70 |
| I66 | 1354.94 | 2.71 | 465.83 | 65 |
| I67 | 554.24 | 1.11 | 736.5 | 52 |
| I68 | 104.65 | 0.08 | 947.67 | 33 |
| I69 | 245.27 | 0.42 | 1056.16 | 36 |
| I70 | 178.67 | 0.20 | 620.4 | 55 |
| I71 | 167.58 | 0.17 | 779.83 | 50 |
| I72 | 423.07 | 0.69 | 200.31 | 36 |
| I73 | 207.13 | 0.25 | 3650.55 | 47 |
| I74 | 348.01 | 0.39 | 4075.04 | 50 |
| I75 | 716.39 | 0.86 | 3609.61 | 53 |
| I76 | 1770.51 | 9.42 | 2141.09 | 67 |
| I77 | 1213.29 | 2.56 | 2199.38 | 75 |
| I78 | 488.39 | 0.85 | 2184.55 | 50 |
| I79 | 1143.58 | 2.63 | 1587.77 | 49 |
| I80 | 1542.19 | 3.07 | 1548.71 | 49 |
| I81 | 576.86 | 0.70 | 1370.77 | 47 |
| I82 | 212.69 | 0.26 | 1175.68 | 36 |
| I83 | 143.56 | 0.12 | 447.66 | 46 |
| I84 | 116.59 | 0.09 | 480.03 | 38 |
| I85 | 3919.17 | 8.98 | 53.8 | 90 |
| I86 | 2726.40 | 10.79 | 2163.04 | 65 |
| I87 | 443.15 | 0.72 | 395.42 | 67 |
| I88 | 186.71 | 0.25 | 431.77 | 44 |
| I89 | 437.10 | 0.97 | 456.31 | 70 |
| I90 | 212.66 | 0.24 | 545.6 | 44 |
| I91 | 157.83 | 0.16 | 552.3 | 38 |
| I92 | 399.09 | 0.69 | 683.47 | 72 |
| I93 | 215.17 | 0.35 | 797.08 | 48 |
| I94 | 1091.32 | 2.59 | 2095.41 | 55 |
| I95 | 1510.62 | 3.73 | 2230.59 | 51 |
| I96 | 724.11 | 1.45 | 1910.16 | 46 |
| I97 | 5040.59 | 38.72 | 1586.83 | 109 |
| I98 | 683.09 | 1.67 | 2345.98 | 66 |
| I99 | 641.32 | 1.88 | 27.87 | 72 |
| I100 | 573.09 | 0.93 | 22.12 | 65 |
| I101 | 1814.56 | 5.57 | 958.07 | 79 |
| I102 | 385.70 | 0.35 | 898.86 | 51 |
| I103 | 118.70 | 0.10 | 880.87 | 32 |
| I104 | 399.03 | 1.20 | 452.58 | 51 |
| I105 | 423.87 | 0.57 | 1238.92 | 68 |
| I106 | 951.60 | 2.02 | 1116.21 | 60 |
| I107 | 536.13 | 1.43 | 1066.9 | 63 |
| I108 | 481.13 | 1.11 | 913.35 | 47 |
| I109 | 8813.28 | 26.78 | 36.2 | 110 |
| I110 | 1107.78 | 2.58 | 1122.2 | 56 |
| I111 | 779.74 | 1.64 | 1226.73 | 42 |
| I112 | 897.52 | 2.04 | 3732.99 | 53 |
| I113 | 542.99 | 0.99 | 4217.1 | 53 |
| I114 | 467.01 | 0.71 | 4067.29 | 60 |
| I115 | 286.58 | 0.43 | 508.81 | 25 |
| I116 | 302.83 | 0.47 | 213.17 | 58 |
| I117 | 3537.63 | 9.79 | 1901.72 | 64 |
| I118 | 128.86 | 0.12 | 696.89 | 46 |
| I119 | 331.74 | 0.26 | 676.31 | 38 |
| I120 | 357.10 | 0.25 | 563.86 | 33 |
| I121 | 100.87 | 0.07 | 393.17 | 30 |
| I122 | 166.47 | 0.20 | 463.7 | 34 |
| I123 | 85.22 | 0.05 | 454.43 | 33 |
| I124 | 1032.02 | 2.29 | 2377.78 | 38 |
| I125 | 3046.34 | 12.40 | 2625.51 | 57 |
| I126 | 2255.26 | 7.99 | 390.61 | 46 |
| I127 | 1212.81 | 4.26 | 63.99 | 45 |
| I128 | 857.50 | 2.24 | 47.36 | 37 |
| I129 | 522.53 | 1.03 | 11.18 | 45 |
| I130 | 457.07 | 1.08 | 106.96 | 45 |
| I131 | 633.83 | 0.97 | 35.35 | 36 |
| I132 | 420.81 | 0.54 | 2319.59 | 55 |
| I133 | 1396.15 | 4.40 | 213.59 | 46 |
| I134 | 1637.21 | 4.67 | 492.12 | 46 |
| I135 | 3020.17 | 9.10 | 30.66 | 55 |
| I136 | 48.40 | 0.02 | 547.16 | 37 |
| I137 | 1604.58 | 4.06 | 48.95 | 62 |
| I138 | 791.03 | 2.39 | 23.81 | 40 |
| I139 | 1036.54 | 2.60 | 15.12 | 44 |
| I140 | 194.64 | 0.22 | 231.97 | 42 |
| I141 | 513.10 | 0.62 | 24.43 | 55 |
| I142 | 298.81 | 0.35 | 26.9 | 54 |
| I143 | 502.35 | 1.07 | 35.09 | 39 |
| I144 | 75.55 | 0.04 | 287 | 46 |
| B4 | 8493.78 | 36.56 | 169.16 | 60 |
| B5 | 11993.60 | 101.04 | 963.55 | 104 |
| B6 | 7523.43 | 47.98 | 1066.1 | 66 |
| B7 | 5622.30 | 27.49 | 1158.87 | 75 |
| B8 | 17550.30 | 130.79 | 55.49 | 80 |
| Longshan | 3357.29 | 35.00 | 993.63 | 143 |
| Xihudi | 7115.73 | 30.99 | 2026.18 | 65 |
| Xikou | 17183.86 | 128.32 | 1417.81 | 116 |
