# Supplementary material for: The Effects of Landscape Variables on the Species-Area Relationship during Late-Stage Habitat Fragmentation
Source: PLoS One. 2012 Aug 24;7(8):e43894. doi: 10.1371/journal.pone.0043894 (PMC3427301; doi:10.1371/journal.pone.0043894)
Supplement: Table S2 — Vascular plant species list on 152 islands in the Thousand Island Lake. (DOCX) [file pone.0043894.s002.docx]

Table S2 Vascular plant species list on 152 study islands in the Thousand Island Lake. Species are listed by alphabetical order of family and species name. Frequency indicates the number of islands the species occurs on.

| **No.** | **Family** | **Species** | **Frequency** |
| --- | --- | --- | --- |
| 1 | Acanthaceae | *Rostellularia procumbens* | 1 |
| 2 | Actinidiaceae | *Actinidia callosa* | 3 |
| 3 | Actinidiaceae | *Actinidia chinensis* | 1 |
| 4 | Adiantaceae | *Adiantum capillus* | 1 |
| 5 | Amaranthaceae | *Alternanthera philoxeroides* | 4 |
| 6 | Amaranthaceae | *Celosia argentea* | 1 |
| 7 | Amaranthaceae | *Chenopodium album* | 1 |
| 8 | Anacardiaceae | *Choerospondias axillaris* | 2 |
| 9 | Anacardiaceae | *Pistacia chinensis* | 6 |
| 10 | Anacardiaceae | *Rhus chinensis* | 114 |
| 11 | Anacardiaceae | *Toxicodendron succedaneum* | 27 |
| 12 | Anacardiaceae | *Toxicodendron sylvestre* | 11 |
| 13 | Apiaceae | *Angelica decursiva* | 1 |
| 14 | Apiaceae | *Centella asiatica* | 120 |
| 15 | Apiaceae | *Cnidium monnieri* | 27 |
| 16 | Apiaceae | *Hydrocotyle sibthorpioides* | 1 |
| 17 | Apiaceae | *Peucedanum praeruptorum* | 1 |
| 18 | Apiaceae | *Torilis scabra* | 8 |
| 19 | Apocynaceae | *Trachelospermum jasminoides* | 1 |
| 20 | Aquifoliaceae | *Ilex chinensis* | 132 |
| 21 | Aquifoliaceae | *Ilex cornuta* | 14 |
| 22 | Aquifoliaceae | *Ilex rotunda* | 11 |
| 23 | Araceae | *Colocasia antiquorum* | 3 |
| 24 | Araliaceae | *Aralia chinensis* | 6 |
| 25 | Araliaceae | *Hedera nepalensis* | 3 |
| 26 | Aspleniaceae | *Asplenium wifordii* | 1 |
| 27 | Asteraceae | *Artemisia anomala* | 1 |
| 28 | Asteraceae | *Artemisia argyi* | 2 |
| 29 | Asteraceae | *Artemisia atrovirens* | 85 |
| 30 | Asteraceae | *Artemisia caruifolia* | 1 |
| 31 | Asteraceae | *Artemisia lavandulaefolia* | 4 |
| 32 | Asteraceae | *Artemisia scoparia* | 14 |
| 33 | Asteraceae | *Aster ageratoides* | 38 |
| 34 | Asteraceae | *Aster panduratus* | 8 |
| 35 | Asteraceae | *Bidens biternata* | 1 |
| 36 | Asteraceae | *Bidens frondosa* | 8 |
| 37 | Asteraceae | *Centipeda minima* | 8 |
| 38 | Asteraceae | *Conyza bonariensis* | 32 |
| 39 | Asteraceae | *Conyza canadensis* | 93 |
| 40 | Asteraceae | *Crassocephalum crepidioides* | 4 |
| 41 | Asteraceae | *Dendranthema indicum* | 17 |
| 42 | Asteraceae | *Erigeron annuus* | 59 |
| 43 | Asteraceae | *Eupatorium chinense* | 19 |
| 44 | Asteraceae | *Eupatorium japonicum* | 3 |
| 45 | Asteraceae | *Gnaphalium affine* | 92 |
| 46 | Asteraceae | *Gnaphalium japonicum* | 1 |
| 47 | Asteraceae | *Hemistepta lyrata* | 12 |
| 48 | Asteraceae | *Ixeridium sonchifolium* | 32 |
| 49 | Asteraceae | *Ixeris polycephala* | 23 |
| 50 | Asteraceae | *Kalimeris indica* | 1 |
| 51 | Asteraceae | *Kalimeris integrifolia* | 1 |
| 52 | Asteraceae | *Paraixeris denticulate* | 4 |
| 53 | Asteraceae | *Pterocypsela formosana* | 16 |
| 54 | Asteraceae | *Pterocypsela indica* | 12 |
| 55 | Asteraceae | *Senecio scandens* | 1 |
| 56 | Asteraceae | *Sinosenecio oldhamianus* | 1 |
| 57 | Asteraceae | *Soliva anthemifolia* | 3 |
| 58 | Asteraceae | *Xanthium sibiricum* | 2 |
| 59 | Asteraceae | *Youngia japonica* | 22 |
| 60 | Blechnaceae | *Woodwardia japonica* | 19 |
| 61 | Boraginaceae | *Bothriospermum zeylanicum* | 1 |
| 62 | Boraginaceae | *Trigonotis peduncularis* | 22 |
| 63 | Brassicaceae | *Cardamine flexuosa* | 7 |
| 64 | Brassicaceae | *Cardamine hirsuta* | 1 |
| 65 | Buxaceae | *Buxus sinica* | 1 |
| 66 | Campanulaceae | *Wahlenbergia marginata* | 48 |
| 67 | Caprifoliaceae | *Abelia chinensis* | 5 |
| 68 | Caprifoliaceae | *Lonicera japonica* | 5 |
| 69 | Caprifoliaceae | *Viburnum dilatatum* | 6 |
| 70 | Caprifoliaceae | *Viburnum erosum* | 5 |
| 71 | Caprifoliaceae | *Viburnum setigerum* | 31 |
| 72 | Caryophyllaceae | *Arenaria serpyllifolia* | 25 |
| 73 | Caryophyllaceae | *Cerastium glomeratum* | 1 |
| 74 | Celastraceae | *Celastrus gemmatus* | 15 |
| 75 | Celastraceae | *Celastrus rosthornianus* | 65 |
| 76 | Celastraceae | *Euonymus carnosus* | 5 |
| 77 | Clusiaceae | *Hypericum japonicum* | 44 |
| 78 | Clusiaceae | *Hypericum patulum* | 4 |
| 79 | Clusiaceae | *Hypericum sampsonii* | 14 |
| 80 | Commelinaceae | *Commelina communis* | 4 |
| 81 | Convolvulaceae | *Calystegia silvatica* | 1 |
| 82 | Cornaceae | *Alangium chinense* | 23 |
| 83 | Cornaceae | *Alangium kurzii* | 18 |
| 84 | Crassulaceae | *Sedum alfredii* | 5 |
| 85 | Crassulaceae | *Sedum bulbiferum* | 17 |
| 86 | Cucurbitaceae | *Zehneria indica* | 1 |
| 87 | Cupressaceae | *Juniperus formosana* | 108 |
| 88 | Cyperaceae | *Carex breviculmis* | 73 |
| 89 | Cyperaceae | *Carex dimorpholepis* | 11 |
| 90 | Cyperaceae | *Carex neurocarpa* | 5 |
| 91 | Cyperaceae | *Cyperus rotundus* | 1 |
| 92 | Cyperaceae | *Fimbristylis dichotoma* | 1 |
| 93 | Cyperaceae | *Mariscus umbellatus* | 2 |
| 94 | Dennstaedtiaceae | *Hypolepis punctata* | 1 |
| 95 | Dioscoreaceae | *Dioscorea japonica* | 1 |
| 96 | Dioscoreaceae | *Dioscorea oppositifolia* | 44 |
| 97 | Dryopteridaceae | *Cyrtomium fortunei* | 2 |
| 98 | Dryopteridaceae | *Dryopteris championii* | 28 |
| 99 | Dryopteridaceae | *Dryopteris fuscipes* | 1 |
| 100 | Ebenaceae | *Diospyros kaki* | 99 |
| 101 | Elaeagnaceae | *Elaeagnus multiflora* | 1 |
| 102 | Elaeagnaceae | *Elaeagnus pungens* | 4 |
| 103 | Elaeocarpaceae | *Elaeocarpus glabripetalus* | 1 |
| 104 | Ericaceae | *Lyonia ovalifolia* | 56 |
| 105 | Ericaceae | *Rhododendron mariesii* | 1 |
| 106 | Ericaceae | *Rhododendron molle* | 3 |
| 107 | Ericaceae | *Rhododendron ovatum* | 32 |
| 108 | Ericaceae | *Rhododendron simsii* | 137 |
| 109 | Ericaceae | *Vaccinium bracteatum* | 119 |
| 110 | Ericaceae | *Vaccinium carlesii* | 141 |
| 111 | Ericaceae | *Vaccinium mandarinorum* | 111 |
| 112 | Escalloniaceae | *Itea oblonga* | 1 |
| 113 | Euphorbiaceae | *Euphorbia helioscopia* | 3 |
| 114 | Euphorbiaceae | *Euphorbia humifusa* | 6 |
| 115 | Euphorbiaceae | *Euphorbia maculata* | 2 |
| 116 | Euphorbiaceae | *Glochidion puberum* | 113 |
| 117 | Euphorbiaceae | *Mallotus apeltus* | 49 |
| 118 | Euphorbiaceae | *Mallotus japonicus* | 16 |
| 119 | Euphorbiaceae | *Mallotus repandus* | 5 |
| 120 | Euphorbiaceae | *Phyllanthus urinaria* | 16 |
| 121 | Euphorbiaceae | *Sapium seibiferum* | 39 |
| 122 | Euphorbiaceae | *Vernicia fordii* | 4 |
| 123 | Euphorbiaceae | *Vernicia montana* | 2 |
| 124 | Fabaceae | *Albizia julibrissin* | 4 |
| 125 | Fabaceae | *Albizia kalkora* | 92 |
| 126 | Fabaceae | *Dalbergia hupeana* | 138 |
| 127 | Fabaceae | *Hylodesmum podocarpium* | 1 |
| 128 | Fabaceae | *Indigofera decora* | 14 |
| 129 | Fabaceae | *Indigofera decora* | 6 |
| 130 | Fabaceae | *Indigofera parkesii* | 7 |
| 131 | Fabaceae | *Indigofera pseudotinctoria* | 20 |
| 132 | Fabaceae | *Kummerowia striata* | 21 |
| 133 | Fabaceae | *Lespedeza bicolor* | 4 |
| 134 | Fabaceae | *Lespedeza buergeri* | 69 |
| 135 | Fabaceae | *Lespedeza cuneata* | 118 |
| 136 | Fabaceae | *Lespedeza davidii* | 3 |
| 137 | Fabaceae | *Lespedeza dunnii* | 11 |
| 138 | Fabaceae | *Lespedeza formosa* | 12 |
| 139 | Fabaceae | *Lespedeza pilosa* | 1 |
| 140 | Fabaceae | *Lespedeza tomentosa* | 7 |
| 141 | Fabaceae | *Millettia dielsiana* | 58 |
| 142 | Fabaceae | *Millettia kiangsiensis* | 3 |
| 143 | Fabaceae | *Millettia reticulata* | 15 |
| 144 | Fabaceae | *Ormosia henryi* | 4 |
| 145 | Fabaceae | *Pueraria lobata* | 1 |
| 146 | Fabaceae | *Rhynchosia volubilis* | 1 |
| 147 | Fabaceae | *Vicia hirsuta* | 3 |
| 148 | Fabaceae | *Vicia sativa* | 1 |
| 149 | Fabaceae | *Vigna vexillata* | 14 |
| 150 | Fabaceae | *Wisteria sinensis* | 17 |
| 151 | Fagaceae | *Castanea henryi* | 1 |
| 152 | Fagaceae | *Castanea mollissima* | 5 |
| 153 | Fagaceae | *Castanopsis eyrei* | 1 |
| 154 | Fagaceae | *Castanopsis jucunda* | 8 |
| 155 | Fagaceae | *Castanopsis sclerophylla* | 78 |
| 156 | Fagaceae | *Cyclobalanopsis glauca* | 17 |
| 157 | Fagaceae | *Cyclobalanopsis gracilis* | 1 |
| 158 | Fagaceae | *Lithocarpus glaber* | 39 |
| 159 | Fagaceae | *Quercus acutissima* | 48 |
| 160 | Fagaceae | *Quercus fabri* | 134 |
| 161 | Fagaceae | *Quercus serrata* | 140 |
| 162 | Geraniaceae | *Geranium carolinianum* | 9 |
| 163 | Gleicheniaceae | *Dicranopteris dichotoma* | 109 |
| 164 | Haloragaceae | *Haloragis micrantha* | 3 |
| 165 | Hamamelidaceae | *Liquidambar formosana* | 77 |
| 166 | Hamamelidaceae | *Loropetalum chinense* | 146 |
| 167 | Illiciaceae | *Illicium lanceolatum* | 1 |
| 168 | Juglandaceae | *Carya cathayensis* | 1 |
| 169 | Juglandaceae | *Juglans regia* | 1 |
| 170 | Juglandaceae | *Platycarya strobilacea* | 12 |
| 171 | Lamiaceae | *Clinopodium gracile* | 3 |
| 172 | Lamiaceae | *Comanthosphace ningpoensis* | 1 |
| 173 | Lamiaceae | *Glechoma longituba* | 34 |
| 174 | Lamiaceae | *Lamium barbatum* | 2 |
| 175 | Lamiaceae | *Leonurus japonicus* | 32 |
| 176 | Lamiaceae | *Salvia plebeia* | 84 |
| 177 | Lamiaceae | *Scutellaria barbata* | 26 |
| 178 | Lamiaceae | *Scutellaria indica* | 29 |
| 179 | Lamiaceae | *Teucrium pernyi* | 1 |
| 180 | Lardizabalaceae | *Akebia quinata* | 2 |
| 181 | Lardizabalaceae | *Sargentodoxa cuneata* | 1 |
| 182 | Lauraceae | *Cinnamomum camphora* | 16 |
| 183 | Lauraceae | *Lindera aggregata* | 86 |
| 184 | Lauraceae | *Lindera erythrocarpa* | 5 |
| 185 | Lauraceae | *Lindera glauca* | 92 |
| 186 | Lauraceae | *Lindera neesiana* | 7 |
| 187 | Lauraceae | *Lindera reflexa* | 1 |
| 188 | Lauraceae | *Lindera rubronervia* | 1 |
| 189 | Lauraceae | *Litsea coreana* | 13 |
| 190 | Lauraceae | *Litsea cubeba* | 10 |
| 191 | Lauraceae | *Machilus leptophylla* | 1 |
| 192 | Lauraceae | *Sassafras tzumu* | 9 |
| 193 | Liliaceae | *Lilium brownii* | 8 |
| 194 | Lindsaeaceae | *Stenoloma chusanum* | 20 |
| 195 | Lygodiaceae | *Lygodium japonicum* | 88 |
| 196 | Lythraceae | *Punica granatum* | 1 |
| 197 | Malvaceae | *Corchoropsis tomentosa* | 30 |
| 198 | Malvaceae | *Grewia biloba* | 2 |
| 199 | Melanthiaceae | *Aletris spicata* | 1 |
| 200 | Melastomataceae | *Melastoma dodecandrum* | 2 |
| 201 | Melastomataceae | *Osbeckia chinensis* | 6 |
| 202 | Meliaceae | *Melia azedarach* | 4 |
| 203 | Menispermaceae | *Cocculus orbiculatus* | 23 |
| 204 | Menispermaceae | *Stephania japonica* | 1 |
| 205 | Moraceae | *Broussonetia kaempferi* | 3 |
| 206 | Moraceae | *Broussonetia kazinoki* | 70 |
| 207 | Moraceae | *Cudrania cochinchinensis* | 1 |
| 208 | Moraceae | *Cudrania tricuspidata* | 6 |
| 209 | Myricaceae | *Myrica rubra* | 13 |
| 210 | Myrsinaceae | *Ardisia japonica* | 3 |
| 211 | Myrtaceae | *Syzygium buxifolium* | 19 |
| 212 | Oleaceae | *Chionanthus retusus* | 9 |
| 213 | Oleaceae | *Ligustrum lucidum* | 1 |
| 214 | Oleaceae | *Ligustrum sinense* | 7 |
| 215 | Orchidaceae | *Cymbidium faberi* | 1 |
| 216 | Osmundaceae | *Osmunda japonica* | 3 |
| 217 | Oxalidaceae | *Oxalis corniculata* | 70 |
| 218 | Oxalidaceae | *Oxalis corymbosa* | 1 |
| 219 | Papaveraceae | *Corydalis pallida* | 1 |
| 220 | Papaveraceae | *Macleaya cordata* | 87 |
| 221 | Phytolaccaceae | *Phytolacca americana* | 11 |
| 222 | Phytolaccaceae | *Phytolacca japonica* | 1 |
| 223 | Pinaceae | *Pinus massoniana* | 146 |
| 224 | Plantaginaceae | *Plantago asiatica* | 7 |
| 225 | Poaceae | *Arthraxon hispidus* | 4 |
| 226 | Poaceae | *Avena fatua* | 1 |
| 227 | Poaceae | *Bromus japonicus* | 4 |
| 228 | Poaceae | *Bromus remotiflorus* | 1 |
| 229 | Poaceae | *Cymbopogon goeringii* | 1 |
| 230 | Poaceae | *Cynodon dactylon* | 1 |
| 231 | Poaceae | *Digitaria ciliaris* | 1 |
| 232 | Poaceae | *Eriochloa villosa* | 69 |
| 233 | Poaceae | *Imperata koenigii* | 19 |
| 234 | Poaceae | *Lophatherum gracile* | 6 |
| 235 | Poaceae | *Miscanthus floridulus* | 141 |
| 236 | Poaceae | *Miscanthus sacchariflours* | 7 |
| 237 | Poaceae | *Miscanthus sinensis* | 33 |
| 238 | Poaceae | *Oplismenus undulatifolium* | 1 |
| 239 | Poaceae | *Panicum bisulcatum* | 1 |
| 240 | Poaceae | *Paspalum thunbergii* | 1 |
| 241 | Poaceae | *Pleioblastus amarus* | 51 |
| 242 | Poaceae | *Poa acroleuca* | 23 |
| 243 | Poaceae | *Poa annua* | 2 |
| 244 | Poaceae | *Polypogon fugax* | 4 |
| 245 | Poaceae | *Roegneria tsukushiensis* | 1 |
| 246 | Poaceae | *Setaria faberii* | 1 |
| 247 | Poaceae | *Setaria viridis* | 18 |
| 248 | Poaceae | *Themeda japonica* | 71 |
| 249 | Poaceae | *Zoysia japonica* | 3 |
| 250 | Polygalaceae | *Polygala hongkongensis* | 106 |
| 251 | Polygalaceae | *Polygala japonica* | 34 |
| 252 | Polygonaceae | *Polygonum hydropiper* | 2 |
| 253 | Polygonaceae | *Polygonum longisetum* | 2 |
| 254 | Polygonaceae | *Polygonum nepalense* | 1 |
| 255 | Polygonaceae | *Polygonum perfoliatum* | 9 |
| 256 | Polygonaceae | *Polygonum pubescens* | 1 |
| 257 | Polygonaceae | *Rumex dentatus* | 2 |
| 258 | Polygonaceae | *Rumex japonicus* | 7 |
| 259 | Primulaceae | *Lysimachia christinae* | 6 |
| 260 | Primulaceae | *Lysimachia clethroides* | 17 |
| 261 | Primulaceae | *Lysimachia congestiflora* | 1 |
| 262 | Primulaceae | *Lysimachia fortunei* | 80 |
| 263 | Primulaceae | *Lysimachia rufopilosa* | 2 |
| 264 | Primulaceae | *Stimpsonia chamaedryoides* | 10 |
| 265 | Pteridaceae | *Pteridium aquilinum* | 95 |
| 266 | Pteridaceae | *Pteris cretica* | 2 |
| 267 | Pteridaceae | *Pteris insignis* | 1 |
| 268 | Pteridaceae | *Pteris multifida* | 29 |
| 269 | Ranunculaceae | *Clematis chinensis* | 2 |
| 270 | Ranunculaceae | *Delphinium anthriscifolium* | 1 |
| 271 | Ranunculaceae | *Ranunculus japonicus* | 27 |
| 272 | Ranunculaceae | *Semiaquilegia adoxoides* | 6 |
| 273 | Rhamnaceae | *Berchemia kulingensis* | 2 |
| 274 | Rhamnaceae | *Rhamnella franguloides* | 4 |
| 275 | Rhamnaceae | *Rhamnus crenatus* | 102 |
| 276 | Rhamnaceae | *Sageretia thea* | 2 |
| 277 | Rosaceae | *Crataegus cuneata* | 14 |
| 278 | Rosaceae | *Duchesnea indica* | 1 |
| 279 | Rosaceae | *Eriobotrya japonica* | 2 |
| 280 | Rosaceae | *Photinia glabra* | 2 |
| 281 | Rosaceae | *Photinia parvifolia* | 21 |
| 282 | Rosaceae | *Photinia serrulata* | 1 |
| 283 | Rosaceae | *Potentilla chinensis* | 8 |
| 284 | Rosaceae | *Potentilla freyrniana* | 5 |
| 285 | Rosaceae | *Potentilla sundaica* | 9 |
| 286 | Rosaceae | *Potentilla supina* | 9 |
| 287 | Rosaceae | *Prunus discoidea* | 7 |
| 288 | Rosaceae | *Prunus grayana* | 9 |
| 289 | Rosaceae | *Prunus persica* | 1 |
| 290 | Rosaceae | *Pyrus calleryana* | 11 |
| 291 | Rosaceae | *Raphiolepis indica* | 113 |
| 292 | Rosaceae | *Rosa bracteata* | 99 |
| 293 | Rosaceae | *Rosa cymosa* | 19 |
| 294 | Rosaceae | *Rosa henryi* | 40 |
| 295 | Rosaceae | *Rosa laevigata* | 127 |
| 296 | Rosaceae | *Rosa multiflora* | 32 |
| 297 | Rosaceae | *Rubus chingii* | 2 |
| 298 | Rosaceae | *Rubus corchorifolius* | 110 |
| 299 | Rosaceae | *Rubus coreanus* | 18 |
| 300 | Rosaceae | *Rubus hirsutus* | 23 |
| 301 | Rosaceae | *Rubus lambertianus* | 5 |
| 302 | Rosaceae | *Rubus rosaefolius* | 1 |
| 303 | Rosaceae | *Spiraea hirsute* | 1 |
| 304 | Rosaceae | *Stephanandra chinensis* | 4 |
| 305 | Rubiaceae | *Galium aparine* | 1 |
| 306 | Rubiaceae | *Galium bungei* | 34 |
| 307 | Rubiaceae | *Gardenia jasminoides* | 139 |
| 308 | Rubiaceae | *Hedyotis chrysotricha* | 97 |
| 309 | Rubiaceae | *Mussaenda shikokiana* | 1 |
| 310 | Rubiaceae | *Paederia scandens* | 34 |
| 311 | Rubiaceae | *Paederia scandens* | 1 |
| 312 | Rubiaceae | *Serissa japonica* | 64 |
| 313 | Ruscaceae | *Liriope muscari* | 1 |
| 314 | Ruscaceae | *Liriope spicata* | 4 |
| 315 | Ruscaceae | *Ophiopogon japonicus* | 7 |
| 316 | Rutaceae | *Poncirus trifoliata* | 1 |
| 317 | Rutaceae | *Zanthoxylum armatum* | 3 |
| 318 | Salicaceae | *Xylosma racemosum* | 2 |
| 319 | Schisandraceae | *Kadsura japonica* | 2 |
| 320 | Scrophulariaceae | *Buddleja lindleyana* | 40 |
| 321 | Scrophulariaceae | *Lindernia procumbens* | 1 |
| 322 | Scrophulariaceae | *Mazus caducifer* | 3 |
| 323 | Scrophulariaceae | *Mazus pumilus* | 11 |
| 324 | Scrophulariaceae | *Monochasma savatieri* | 57 |
| 325 | Scrophulariaceae | *Paulownia fortunei* | 1 |
| 326 | Scrophulariaceae | *Siphonostegia laeta* | 2 |
| 327 | Scrophulariaceae | *Veronica peregrine* | 1 |
| 328 | Scrophulariaceae | *Veronica persica* | 5 |
| 329 | Scrophulariaceae | *Veronica undulata* | 12 |
| 330 | Selaginellaceae | *Selaginella nipponica* | 1 |
| 331 | Simaroubaceae | *Ailanthus altissima* | 1 |
| 332 | Smilacaceae | *Smilax arisanensis* | 2 |
| 333 | Smilacaceae | *Smilax china* | 135 |
| 334 | Smilacaceae | *Smilax davidiana* | 72 |
| 335 | Smilacaceae | *Smilax glabra* | 109 |
| 336 | Solanaceae | *Physalis angulata* | 2 |
| 337 | Solanaceae | *Solanum lyratum* | 1 |
| 338 | Solanaceae | *Solanum nigrum* | 3 |
| 339 | Staphyleaceae | *Euscaphis japonica* | 10 |
| 340 | Styracaceae | *Alniphyllum fortunei* | 9 |
| 341 | Styracaceae | *Pterostyrax corymbosa* | 1 |
| 342 | Styracaceae | *Styrax dasyanthus* | 17 |
| 343 | Styracaceae | *Styrax faberi* | 54 |
| 344 | Styracaceae | *Styrax odoratissimus* | 30 |
| 345 | Symplocaceae | *Symplocos paniculata* | 113 |
| 346 | Symplocaceae | *Symplocos setchuensis* | 1 |
| 347 | Symplocaceae | *Symplocos stellaris* | 54 |
| 348 | Symplocaceae | *Symplocos sumuntia* | 65 |
| 349 | Taxodiaceae | *Cunninghamia lanceolata* | 43 |
| 350 | Theaceae | *Camellia chekiang* | 4 |
| 351 | Theaceae | *Camellia fraterna* | 65 |
| 352 | Theaceae | *Camellia sinensis* | 6 |
| 353 | Theaceae | *Cleyera japonica* | 12 |
| 354 | Theaceae | *Eurya muricata* | 117 |
| 355 | Theaceae | *Schima superba* | 18 |
| 356 | Thymelaeaceae | *Wikstroemia monnula* | 12 |
| 357 | Thymelaeaceae | *Wikstroemia pilosa* | 1 |
| 358 | Ulmaceae | *Aphananthe aspera* | 2 |
| 359 | Ulmaceae | *Celtis biondii* | 1 |
| 360 | Ulmaceae | *Trema cannabina* | 63 |
| 361 | Ulmaceae | *Ulmus parvifolia* | 20 |
| 362 | Urticaceae | *Boehmeria clidemioides* | 1 |
| 363 | Urticaceae | *Boehmeria nivea* | 4 |
| 364 | Urticaceae | *Pilea peploides* | 2 |
| 365 | Urticaceae | *Pilea pumila* | 1 |
| 366 | Urticaceae | *Pouzolzia zeylanica* | 1 |
| 367 | Valerianaceae | *Patrinia scabiosaefolia* | 88 |
| 368 | Verbenaceae | *Callicarpa bodinieri* | 4 |
| 369 | Verbenaceae | *Callicarpa cathayana* | 3 |
| 370 | Verbenaceae | *Callicarpa giraldii* | 9 |
| 371 | Verbenaceae | *Callicarpa rubella* | 2 |
| 372 | Verbenaceae | *Caryopteris incana* | 4 |
| 373 | Verbenaceae | *Clerodendrum cyrtophyllum* | 3 |
| 374 | Verbenaceae | *Premna microphylla* | 51 |
| 375 | Verbenaceae | *Vitex negundo* | 94 |
| 376 | Violaceae | *Viola concordifolia* | 2 |
| 377 | Violaceae | *Viola diffusa* | 13 |
| 378 | Violaceae | *Viola philippica* | 110 |
| 379 | Vitaceae | *Ampelopsis heterophylla* | 8 |
| 380 | Vitaceae | *Ampelopsis japonica* | 2 |
| 381 | Vitaceae | *Cayratia japonica* | 1 |
| 382 | Vitaceae | *Vitis hancockii* | 11 |
| 383 | Vitaceae | *Vitis pseudoreticulata* | 9 |
